# Le stress perçu: validation de la traduction d'une échelle de mesure de stress en dialecte marocain

**DOI:** 10.11604/pamj.2015.21.280.6430

**Published:** 2015-08-12

**Authors:** Dalal Ben Loubir, Zeineb Serhier, Nada Otmani, Samy Housbane, Naima Ait Mouddene, Mohamed Agoub, Mohammed Bennani Othmani

**Affiliations:** 1 Laboratoire d'Informatique Médicale, Faculté de Médecine et de Pharmacie, Université Hassan 2, Casablanca, Maroc; 2 Laboratoire de Neurosciences et Santé Mentale, Faculté de Médecine et de Pharmacie, Université Hassan 2, Casablanca, Maroc

**Keywords:** Perceived stress scale, traduction, validation, dialecte Marocain, Perceived stress scale, traduction, validation, Moroccan dialect

## Abstract

**Introduction:**

L'article a pour objectif de valider la traduction en dialecte arabe Marocain, de l’échelle du stress perçu « Perceived Stress Scale » chez un large échantillon de la population marocaine.

**Méthodes:**

Une méthode de traduction et contre traduction en dialecte Marocain a été réalisée pour l’échelle « Perceived Stress Scale » à 10 items. Une étude de type transversale, a été menée par la suite en suivant un échantillonnage par quotas qui a ciblé les individus âgés de plus de 18 ans et appartenant à différentes catégories sociales. Les participants ont répondus aux versions marocaines de l’échelle « Perceived Stress Scale » et d'une version réduite de l’échelle « The Survey of Recent Life Experience ». La validation des qualités métrologiques a été établie en examinant: la fiabilité interne, la fiabilité test-retest, la validité convergente et la structure factorielle.

**Résultats:**

Cinq cent trente-cinq participants ont répondu aux questionnaires. La version marocaine de l’échelle « Perceived Stress Scale » a présenté une bonne fiabilité interne (α = 0,80) et fiabilité test-retest (CCI= 0,87). D'autant plus, l'analyse en composante principale de l’échelle « Perceived Stress Scale » a permis d'extraire deux facteurs qui ont permis d'expliquer 53,4% de la variance totale. Les deux échelles utilisées étaient assez bien corrélées (r = 0,467, p < 0,01).

**Conclusion:**

La présente étude a permis d'obtenir une version marocaine de l’échelle « Perceived Stress Scale » avec des propriétés psychométriques satisfaisantes, dont l'utilisation sera d'une grande aide dans la pratique médicale, aussi bien que dans la réalisation des études de recherche qui s'intéressent au stress psychologique.

## Introduction

L'intérêt scientifique et médical pour le stress psychologique est assez ancien, il a permis d'instaurer une littérature abondante qui aborde le thème de la mesure du stress psychologique et qui rapporte les résultats des enquêtes, sur l'association du stress psychologique et une variété de problèmes sanitaires.

Pour définir le stress psychologique, Lazarus et Folkman [1] ont proposé en 1991 un modèle transactionnel qui stipule que: « le stress psychologique est une relation particulière entre la personne et son environnement, évaluée par celle-ci comme excédant ses capacités et mettant en danger son bien-être ». Ce modèle transactionnel repose sur la prise en considération de l'interaction qui existe entre l'individu et son environnement: l'individu n'admet pas passivement la situation mais il opte pour diverses stratégies cognitives, affectives, comportementales et psychosociales pour y faire face. Les stratégies développées par l'individu en réponse à une situation stressante, peuvent ralentir ou accélérer l’évolution du processus pathologique. Ainsi donc, la psychologie de la santé prend en charge l’étude du rôle médiateur de ces transactions (stress perçu, contrôle perçu, soutien social, coping…) capables de moduler l'apparition ou bien l’évolution d'une maladie [2].

Sur la base de la théorie transactionnelle, Cohen et ses collègues en 1983 [3] ont développé une échelle auto-administrée, « Perceived Stress Scale » à 14 items (PSS14), qui mesure la perception des situations stressantes par l'individu. Par la suite, une version réduite a été extraite, PSS10 à dix items, qui a conservé les items qui ont présenté des saturations élevés à l'analyse factorielle [4]. L’évaluation de l’échelle PSS10 a montré qu'elle possède des propriétés psychométriques adéquates proches de la version originale PSS14, et grâce au nombre réduit de ses items, PSS10 a été largement utilisée. Plusieurs études ont montré que cette échelle était positivement corrélée aux mesures de la symptomatologie psychologique et physiologique [5–9]. Ainsi, l’échelle PSS10 a fait l'objet de diverses traductions et validation en plusieurs langues [10–12], y compris la langue Arabe Classique [13, 14]. Bien que le Maroc soit un pays Arabe qui utilise la langue Arabe classique comme première langue officielle dans les administrations et les écoles, et bien que les marocains apprennent cette langue au cours de leur jeune âge dans les écoles, le dialecte marocain reste la langue parlée et comprise par la majorité de la population marocaine.

Le but de notre étude était de traduire et de valider l’échelle PSS10 en dialecte arabe Marocain, afin de mettre en place un outil de mesure de stress psychologique en dialecte local adéquat pour l'ensemble de la population marocaine.

## Méthodes

### Traduction

La traduction de l’échelle PSS10 de l'anglais vers le dialecte arabe marocain (PSS10d) a été réalisée par trois traducteurs différents. Les versions obtenues ont été synthétisées lors d'une réunion afin d'obtenir une seule version. Ensuite, cette dernière a été rétro-traduite vers la langue originale (anglais), par deux traducteurs non familiarisées avec l’échelle PSS10. La clarté des items et l'absence de différences avec la version originale ont été vérifiées par un comité englobant les traducteurs et des psychiatres. Afin d’éliminer toute ambigüité et pour vérifier la compréhension des différents items, un test a été réalisé chez un groupe de 10 individus appartenant à différentes catégories sociales et niveaux d’éducation.

### Collecte et analyse des données

Nous avons mené une étude transversale entre Avril et Juillet 2014. Un échantillonnage par quotas ayant comme critère l'alphabétisation et le sexe a été utilisé. Les participants marocains, parlant le dialecte marocain et dont l’âge était supérieur à 18 ans, étaient invités à prendre part à l’étude. L’échantillon était composé: des étudiants de la Faculté de Médecine et de Pharmacie de Casablanca (FMPC); du corps administratif de la FMPC; des visiteurs dans certains services hospitaliers du CHU Ibn Rochd à Casablanca; des familles et des amis des enquêteurs.

Tous les participants ont été informés sur l'objectif de l’étude, et sur l'anonymat et la confidentialité des données recueillies. Un questionnaire anonyme auto-administré, en dialecte marocaincontenant 38 questions a été distribué aux participants après avoir obtenu leur consentement. Pour les participants analphabètes, les questionnaires ont été administrés par les enquêteurs.

Le questionnaire se composait de la version marocaine de l’échelle de mesure du stress perçu PSS10d, dont les réponses pour chaque item varient selon une échelle de Likert à cinq points allant de 0 (jamais) à 4 (très souvent), et qui évaluent le niveau du stress perçu durant le dernier mois [4]. L’échelle PSS10 est composée de deux facteurs, le premier regroupe six items négatifs mesurant « la perception du stress » chez l'individu, alors que le deuxième regroupe quatre items positifs, mesurant le « coping » ou l'adaptation au stress [4]. Le score total du stress est obtenu par la somme des différentes réponses après inversion des items positifs, et il varie de 0 à 40. Un score élevé indique un niveau élevé de stress perçu. Pour évaluer la validité convergente de PSS10d, vingt-deux items de l’échelle «The Survey of Recent Life Experiences » (SRLE) [15], ont été sélectionnés afin de vérifier la corrélation entre les scores de PSS10d et ces items. Ces 22 items qui forment l’échelle «The Survey of Recent Life Experiences » réduite (SRLEr), ont été choisis sur la base de leur adaptation au contexte marocain. L’échelle SRLE mesure le degré des tracas quotidiens expérimentés par le participant. Celui-ci est invité à évaluer, la fréquence des fois où il a été préoccupé, durant le mois passé, à cause d'un évènement particulier. Les réponses sont données sur une échelle de Likert à quatre points (1 = ne fais pas du tout partie de ma vie à 4= fait partie intégrante de ma vie). Le score total de SRLEr variait de 22 à 88. Ainsi, des scores élevés indiquent des fréquences élevés de tracas de vie récents. L’échelle SRLE a présenté des propriétés psychométriques adéquates au cours de son utilisation, d'autant plus,elle est corrélée à diverses mesures de la symptomatologie psychologique [16]. Les 22 items de SRLEr ont été traduits en dialecte marocain puis ont été distribués chez un groupe de six personnes pour vérifier la clarté des items. D'autres variables ont été incluses dans le questionnaire, tel que le sexe, l’âge, l’état matrimonial, la profession etle tabagisme.

Pour apprécier la reproductibilité de l’échelle PSS10d, lors de la collecte des données un petit échantillon incluant les employés de la FMPC a été sollicité à participer au retest, en rendant chaque questionnaire personnel nominatif. Cinquante-trois participants ont accepté de participer au retest, et ce en complétant à nouveau l’échelle PSS10d dans un intervalle de temps de 24 heures.

Durant l'analyse des données, pour les individus qui ne travaillent pas: étudiants et non-actif (femmes au foyer, retraités et chômeurs), le calcul du score moyen de SRLEr a été effectué en omettant les scores de la dimension « Travail », qui comporte quatre items. De ce fait, le score moyen de SRLEr, pour ces deux catégories variait de 18 à 62. Ainsi pour pouvoir comparer les scores de SRLEr dans l’échantillon en sa totalité, les scores de SRLEr ont été normalisés pour varier de 0 à 100 ((score-min/étendu)*100; le score min est de 18 pour les catégories « non-actifs » et « étudiants », et est 22 pour les autres catégories). Les scores ont été décrits par leur moyenne et écart-type (ET).

La fiabilité interne des deux échelles a été mesurée par le coefficient alpha de Cronbach, et le coefficient de corrélation intra-classe (CCI) a été utilisé afin d'examiner la fiabilité test-retest de l’échelle PSS10d. L'examen de la validité du construit de PSS10d était basé sur l'analyse en composante principale avec rotation Varimax. Les facteurs avec des valeurs propres supérieurs à 1 ont été retenus. L'examen de la validité convergente a fait appel aux coefficients de corrélation de Spearman entre l’échelle PSS10d et l’échelle SRLEr. Toutes les analyses ont été effectuées en utilisant lelogiciel SPSS 16.0. L’étude a été approuvée par le «COMITE D'ETHIQUE POUR LA RECHERCHE BIOMEDICALE CASABLANCA » de la faculté de Médecine et de Pharmacie de Casablanca.

## Résultats

Cinq cent trente-cinq participants ont répondu aux questionnaires (taux de réponse: 97%), dont 50,8% était de sexe féminin et 25% était analphabètes. La moyenne d’âge de l'ensemble de l’échantillon était de 37,3 ans (±14,5). Près de la moitié des participants (49,6%) étaient mariés, et plus de 29% des participants étaient fumeurs. Les caractéristiques démographiques de l'ensemble de l’échantillon sont présentées dans le Tableau 1.

**Tableau 1:** Caractéristiques démographiques de l’échantillon étudié (N = 535)

Caractéristiques		%
Age (année)	≤ 35	52,3
	>35	47,7
Genre	Féminin	50,8
	Masculin	49,2
Profession	Etudiant	20,2
	Actif	41,8
	Non-actif	35,8
	Non-cité	2,2
Niveau d’éducation	Analphabète	25,0
	Primaire	11,0
	Secondaire	24,5
	Universitaire	39,4
Situation sociale	Célibataire	40,3
	Marié	49,6
	Divorcé/veuf	10,1
Tabagisme	Oui	29,3
	Non	70,7

Concernant les scores de PSS10d, une moyenne de 20,2 (ET = 6,2) a été rapportée chez les répondants, avec des scores légèrement élevés chez le sexe féminin (21,1 (ET = 6,1)), chez les répondants appartenant à la catégorie « non-actif » (21,3 (ET = 6,1)) et chez la catégorie des analphabètes (21,2 (ET = 5,2)). De même, pour l’échelle SRLEr, un score moyen de 45,5 (ET = 16,7) a été rapporté chez l'ensemble des répondants. Le Tableau 2 résume les scores de stress obtenus par les échelles PSS10d et SRLEr dans l’échantillon étudié. La fiabilité interne de PSS10d et SRLEr a été bonne avec un coefficient α = 0,80 pour les deux échelles. Concernant la reproductibilité test-retest de l’échelle PSS10d, le CCI= 0,87 (IC_95%_: [0,77-0,92]).

**Tableau2:** Associations des scores de PSS10d et SRLEr avec les variables étudiées

Variables	PSS10d Moyenne (ET)		p	SRLEr Moyenne (ET)	p
Genre			<0,01		<0,05
	Masculin	19,2 (6,2)		43,8 (16,6)	
	Féminin	21,1 (6,1)		47,2 (16,7)	
Situation sociale			0,54		0,07
	Célibataire	20,4 (6,4)		43,7 (17,4)	
	Marié	19,9 (6,1)		46,2 (16,4)	
	Divorcé/veuf	20,7 (6,3)		48,98 (14,5)	
Niveau d’étude			0,05		<0,01
	Analphabète	21,2 (5,2)		49,1 (15,4)	
	Primaire	21,1 (6,7)		49,6 (17,0)	
	Secondaire	19,3 (6,0)		42,4 (17,7)	
	Universitaire	19,8 (6,7)		44,0 (16,3)	
Profession			<0,01		<0,01
	Etudiant	20,5 (6,3)		43,3 (16,6)	
	Non actif	21,3 (6,1)		51,3 (16,7)	
	Actif	19,3 (6,1)		42,4 (15,4)	
Tabagisme			0,23		<0,05
	Oui	20,7 (6,4)		48,1 (16,7)	
	Non	19,9 (6,1)		44,4 (16,6)	
Echantillon Total		20,2 (6,2)		45,5 (16,7)	

ET : Ecart-type

Le coefficient de corrélation obtenu entre les échelles PSS10d et SLREr (r = 0,467; p < 0,01) révèle que ces deux échelles sont assez bien corrélées. Toutefois, les coefficients de corrélation obtenus dans le Tableau 3, montrent des associations significatives entre PSS10d et les dimensions de SRLEr mais qui sont faibles. Pour ce qui est de l'analyse en composante principale, il faut noter que la valeur de KMO et le test de la sphéricité de Bartlett étaient significatifs dans l’échantillon étudié, suggérant ainsi que les résultats sont acceptables. Comme le montrent les résultats dans le Tableau 4, l'analyse en composante principale a permis d'obtenir deux facteurs ayant des valeurs propres supérieurs à 1. L'extraction de ces deux facteursa permis d'expliquer 53,4% de la variance totale des items dans l’échantillon étudié. Dans ce modèle bi-structurel, le facteur 1 expliquait la variabilité des items liés au stress négatif (items: 1, 2, 3, 6, 9, 10), alors que le facteur 2 expliquait la variabilité des items liés à l'adaptation au stress (items: 4, 5, 7, 8).

**Tableau 3:** Corrélations entre PSS10d et les dimensions de SRLEr

Dimensions	Difficultés sociales et culturelles	Travail	Argent	Temps	Acceptabilité social	Victimisation sociale
PSS10d	0,364^a^	0,333^a^	0,378^a^	0,169	0,356^a^	0,199^a^
Alpha de Cronbach	0,50	0,66	0,64	0,59	0,70	0,50

a Corrélation significative au niveau 0,01

**Tableau 4:** Résultats de l'analyse en composante principale avec rotation Varimax

Items de PSS10d	Facteur1	Facteur2
1. être dérangé à cause d'un événement inattendu	0,782	-
2. croire qu'il est difficile de contrôler les choses importantes de la vie	0,706	-
3. se sentir nerveux ou stressé	0,768	-
4. se sentir confiant à prendre en mains les problèmes personnels	-	0,758
5. avoir le sentiment que les choses aillent dans le bon sens	-	0,688
6. penser ne pas pouvoir assumer toutes les choses qui doivent être faites	0,630	-
7. être capable de maîtriser son énervement	-	0,700
8. avoir le sentiment de dominer la situation	-	0,713
9. se sentir irrité à cause des événements qui échappent au contrôle	0,700	-
10. sentir les difficultés s'accumuler à un tel point qu'il devient impossible de les contrôler	0,600	-
Valeur de KMO	0,838	
Sphéricité de Bartlett	<0,0001	
Valeurs propres	3,6	1,7
% de variance expliquée	36,4	17,0
	53,4	

Les valeurs inférieures à 0,30 ont été supprimées

## Discussion

Les résultats de la présente étude montrent que l’échelle PSS10d présente des propriétés psychométriques satisfaisantes. En concordance avec la littérature [11, 17, 18], notre étude a révélé que les scores de PSS10d sont associés au sexe et à la profession (p < 0.01). En effet; les femmes, les étudiants et les répondants appartenant à la catégorie des « non-actifs » présentent des scores moyennement élevé de PSS10d par rapport aux autres catégories. Ces différentes associations ont été confirmées par les scores de SRLEr qui ont été élevés chez les catégories précédemment mentionnées. Les scores de stress variaient aussi avec le niveau d’éducation et la situation maritale, comme a été rapporté dans d'autres études [11, 18]. Dans notre échantillon, les répondants appartenant à la catégorie « universitaire », et les répondants appartenant à la catégorie « marié » étaient moins stressés que les répondants appartenant aux catégories complémentaires. Néanmoins, L'association des scores de PSS10d avec le niveau d’éducation n’était pas statistiquement significative.

Concernant les propriétés psychométriques de l’échelle PSS10d, celle-ci a présenté une fiabilité interne satisfaisante qui était proche de la fiabilité interne obtenue pour la version originale de PSS10 (α = 0.78) [4] et proche aussi de la fiabilité interne obtenue pour la version Arabe classique de PSS10 établie précédemment (α = 0.74) par Chaaya et al [13]. La reproductibilité de PSS10d après 24 heures de ré-administration a été satisfaisante (CCI= 0,87).

Une corrélation positive et significative a été observée pour les scores de PSS10d et SRLEr qui rejoint les résultats de la littérature [15]. Cependant les coefficients de corrélation obtenus étaient faibles. Ceci pourrait être expliqué par le fait qu'un score élevé de SRLEr (correspondant à une fréquence élevée des événements stressants) peut être associé à un score élevé de stress (PSS10d). Néanmoins, certains individus qui possèdent d'assez bonnes méthodes d'adaptation avec les événements stressants peuvent rapporter des scores moyens de PSS10d, malgré la confrontation fréquente aux événements stressants (scores élevés de SRLEr).

Le résultat obtenu pour la structure factorielle est en concordance avec celui trouvé dans la version originale de PSS10 [4], et avec la littérature qui a concerné la traduction et la validation de PSS en différentes langues. La structure bi-factorielle de PSS, qui présente le premier facteur regroupant les items négatifs et le deuxième facteur regroupant les items positifs, a été largement observée dans les études de traduction de PSS10 [13, 19]. Selon Hewitt et al [20] le premiers facteur est nommé « stress perçu », car il inclut les items qui se rapportent aux réactions affectives négatives. Tandis que le deuxième facteur, noté « coping », regroupe les items reflétant la perception de la capacité d'adaptation aux événements stressants. Concernant la variance totale expliquée, celle-ci a été très satisfaisante dans notre échantillon.

Il faut noter aussi que l’échelle SRLEr utilisée a présenté des propriétés psychométriques satisfaisantes en terme de fiabilité. L’échelle SRLEr a montré les mêmes associations significatives, que l’échelle PSS10d, avec le sexe et la profession, bien qu'elle n'a inclus qu'un nombre réduit de ses items. L'utilisation de la totalité des items de l’échelle SRLE dans le questionnaire employé dans notre étude aurait été difficile, car celle-ci pouvait influencée le taux de réponse des participants qui peuvent refuser de répondre lorsqu'il s'agit de questionnaire très long.

D'un autre côté, bien que l’échantillon de notre étude fût hétérogène, la méthode d’échantillonnage entretenue dans notre étude ne permet pas de généraliser les résultats du stress perçu sur toute la population Marocaine. Il est donc difficile de conclure qu'une catégorie sociodémographique spécifique est plus stressée qu'une autre.

## Conclusion

En conclusion, la présente étude a permis d'obtenir une version marocaine de l’échelle PSS10 avec des propriétés psychométriques satisfaisantes, dont l'utilisation pourrait apporter plusieurs avantages dans la pratique médicale, aussi bien que dans la réalisation des études de recherche s'intéressant au stress psychologique. En outre, vu que c'est une échelle courte à 10 items, PSS10d peut être utilisée dans les situations qui favorisent les échelles courtes, tels que les enquêtes téléphoniques ou électroniques, pour mesurer le stress perçu au sein de la population marocaine.
